# Clinical investigation of use of Episil® oral solution in oral mucositis during radiotherapy for head and neck cancer

**DOI:** 10.1016/j.heliyon.2023.e15869

**Published:** 2023-05-24

**Authors:** Kanade Ito, Shiori Tokura, Itsuki Takazawa, Naomi Yoshida, Tohko Nakanishi, Kikue Akiyama, Yuki Onuma, Toshiko Adachi, Hiroyuki Harada, Hitomi Nojima, Masahiko Miura, Ryoichi Yoshimura, Yuji Kabasawa

**Affiliations:** a Department of Oral Care for Systemic Health Support, Graduate School of Medical and Dental Sciences, Tokyo Medical and Dental University, Japan; b Department of Oral Health Care Education, Graduate School of Medical and Dental Sciences, Tokyo Medical and Dental University, Japan; c Department of Dental Hygiene, Tokyo Medical and Dental University Hospital, Japan; d Department of Oral and Maxillofacial Surgical Oncology, Graduate School of Medical and Dental Sciences, Tokyo Medical and Dental University, Japan; e Department of Dental Radiology and Radiation Oncology, Graduate School of Medical and Dental Sciences, Tokyo Medical and Dental University, Japan; f Department of Radiation Therapeutics and Oncology, Graduate School of Medical and Dental Sciences, Tokyo Medical and Dental University, Japan

**Keywords:** Episil®, Head and neck cancer, Oral mucositis, Radiotherapy

## Abstract

**Objective:**

Episil® is a bio adhesive barrier-forming oral liquid gel that has been used in recent years to relieve pain of oral mucositis (OM) with radiotherapy (RT) or chemoradiotherapy (CRT) in head and neck cancer (HNC) patients. We conducted a retrospective analysis of the clinical effects of Episil® on OM in these patients.

**Study design:**

Between June 2018 and May 2020, 65 patients with HNC were treated with RT or CRT at our hospital.

**Results:**

The median total RT dose was 50 Gy (range, 30–70 Gy) and the completion rate was 63/65 (97%). The median time to OM resolution was 47 (6–90) days and was significantly longer (53 [27–90] days) when the total RT dose was ≥51 Gy (*P* < 0.001). Episil® was used in 26 patients. Among them, 10 discontinued its use due to ineffective pain relief, usage difficulties, and taste intolerance. The median duration of use was 30 days and was significantly longer (34.5 days) (*P* < 0.001) when patients experienced pain relief at treatment initiation.

**Conclusion:**

Although Episil® has been shown to be effective in improving the pain of OM caused by RT for HNC patients, and medical professionals are required to give careful attention to each patient.

## 1 Introduction

**Fig. 1:**
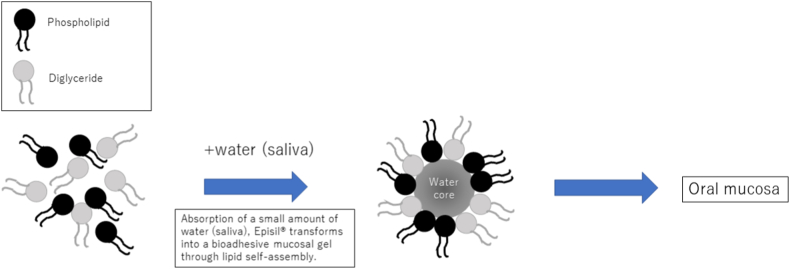
Mechanism of action of Episil®. When applied to the oral mucosa, the phospholipid (soybean phosphatidylcholine) and diglyceride (caprylic acid diglyceride) components self-assemble with trace amounts of water (saliva) to form a bio adhesive liquid lining that protects the oral mucosa. (Figure is modified from Hadjieva et al. [8].)

Oral mucositis (OM) is the primary complication of radiotherapy (RT) or chemoradiotherapy (CRT), causing decreased quality of life (QOL) in patients with head and neck cancer (HNC) [[1], [2], [3]]. Additionally, it can cause decreased RT or CRT completion rates, leading to poor clinical outcomes in these patients [4]. The Mucositis Study Group of the Multinational Association of Supportive Care in Cancer (MASCC)/International Society for Oral Oncology (ISOO) clinical practice guidelines [5,6] recommend the use of several prophylactic, therapeutic, and pain control methods for OM during RT or CRT, such as oral cryotherapy, human keratinocyte growth factor-1 (KGF-1/palifermin), low-level laser therapy (LLLT), patient-controlled analgesia with morphine, benzydamine mouthwash, transdermal fentanyl, 2% morphine mouthwash, 0.5% doxepin mouthwash, and zinc supplements. Additionally, they recommend basic oral care, including a combination of tooth brushing, flossing, and mouth rinsing at least once daily (with an alcohol-free, saline and sodium bicarbonate mouthwash) to maintain oral hygiene [7]. However, in Japan, KGF-1/palifermin, LLLT, benzydamine mouthwash, transdermal fentanyl, 2% morphine mouthwash, and 0.5% doxepin mouthwash are not approved for use and are not covered by medical insurance. In contrast, since 2018, to optimize the oral health care of cancer patients, Episil® has been the only bio adhesive barrier-forming oral liquid that is covered by medical insurance and available for use in Japan. In Japan, dentists prescribe Episil® in collaboration with physicians to manage the pain experienced during OM in patients with cancer. Episil® is a lipid-based drug carrier system that is used in the oral cavity and it contains soybean lecithin and diolein. When it comes in contact with the oral mucosa, oil accumulates on the surface of the saliva and forms small spheres that join rapidly to form a thin gel skeleton arrangement, which creates a physical barrier. The lipid and ambient water components in the saliva undergo a non-chemically mediated molecular self-assembly to form lipid films (Fig. 1) [8].

Episil® should be swirled around the mouth approximately 1 h before eating to form a protective layer over the painful areas. Additionally, topical analgesics may be co-administered to intensify the therapeutic effect. It should be noted that Episil® and the topical analgesics should not be swallowed. Episil® adheres to ulcerative areas and within 5 min forms a protective membrane that acts as a mechanical barrier, relieving pain. Additional effects of the bio adhesive lipid formation include lubrication, moisturization, and mechanical protection of the sore mucosa.

Although some bio adhesive barrier-forming oral liquids do provide comfort for patients, due to the insufficient evidence with regard to their ability to reduce the severity of mucositis, the current MASCC/ISOO guidelines do not mention the effectiveness of these agents [9]. The European Oral Care in Cancer Group and UK Oral Mucositis in Cancer Group have highlighted the usefulness of topical gel or film oral mucosal protectants, such as Caphosol®, Mugard®, Oralife®, Gelclair®, and Episil®, and have recommended their use for mild to moderate OM [10].

While previous studies have reported that Episil® is an effective and safe product when used for the relief of pain caused by OM in patients with cancer [8,11], these did not demonstrate clearly, the efficacy of Episil® when used alone. Ueno T et al. used Episil® in ten Japanese cancer patients and clearly demonstrated its pain-relieving effect on OM [12], however, only one RT with HNC patient was included, the effects of Episil® in patients with HNC, remain unclear. Moreover, adverse reactions to Episil® have been observed, including nausea and difficulties experienced in the proper application at the OM site [13]. Clinically, we have observed both benefits and difficulties when using Episil® in patients with HNC; however, these experiences have been rarely discussed in the literature and thus, more evidence is needed to support the use of Episil® in patients with HNC. Therefore, we aimed to examine the pain-relieving effects and patients' perceptions of Episil® during the initiation of RT or CRT for HNC.

## 2 Materials and methods

This was a retrospective study that examined the clinical data of 65 patients with HNC who underwent RT or CRT at our hospital between June 2018 and May 2020. Among the 65 tumors, 64 were histologically confirmed as squamous cell carcinomas, and one as malignant lymphoma (Table 1).

**Table 1:** Baseline characteristics of the Episil® use group and control group (n = 65).

Characteristic	Use (n = 26)	Control (n = 39)	*P* value
**Age (y)** Median (min–max)	62.5 (41–83)	68 (22–90)	0.076
**Irradiation total (Gy)**			
Median (min–max)	54 (30–66)	50 (40–70)	0.897
**Irradiation site**			
Oral cavity	10	20	
Oral cavity + neck	10	16	
Oropharynx	0	1	
Oropharynx + neck	2	0	
Neck	4	2	
			0.371
**Method of RT**			
3D-CRT	16	30	
IMRT	10	9	
			0.367
**Concurrent chemotherapy**			
CDDP	11	21	
TS-1	13	14	
Cmab	1	3	
None	1	1	
			0.536

RT: radiotherapy; CDDP: cisplatin; 3D-CRT: three-dimensional conformal radiotherapy; IMRT: intensity-modulated radiotherapy; TS-1: tegafur-gimeracil-oteracil-potassium; Cmab: cetuximab.

All the patients received RT using three-dimensional conformal RT (3D-CRT) (46 patients) or intensity-modulated RT (IMRT) (19 patients). The overall therapeutic irradiation dose was 30–70 Gy (median, 50 Gy). The purpose of RT was post-operative irradiation. Two patients had their planned RT doses reduced and interrupted (30 Gy and 54 Gy) due to a deterioration of their nutritional status. The original dose for a 30 Gy patient was 50 Gy, and the original dose for a 54 Gy patient was 60 Gy. RT was performed once a day (2 Gy) five times a week. In addition, 63 patients received concurrent chemotherapy; the regimens included either cisplatin (CDDP; 30 mg/m^2^/w), tegafur-gimeracil-oteracil-potassium (TS-1®) (80 mg/m^2^), or cetuximab (400 mg/m^2^/w and 250 mg/m^2^/w) (Table 2).

**Table 2:** Relationship of various factors with days until resolution of OM (n = 65).

	n	Days to Resolution of OM Median (min–max)	*P* value
Total	65	47 (6–90)	
Episil®			
Use	26	43 (12–90)	
Control	39	49 (6–88)	0.312
Mucositis grade			
1	4	31 (24–39)	
2	21	37 (12–90)	
3	37	51 (6–88)	
4	3	49 (41–53)	
			0.407^＊^
Irradiation total (Gy)			
30–50	33	39 (6–57)	
51–70	32	53 (27–90)	
			<0.001
Irradiation site			
Oral cavity	30	46 (6–74)	
Oral cavity + neck	26	49 (12–90)	
Oropharynx	1	68	
Oropharynx + neck	2	53 (51–54)	
Neck	6	39 (27–40)	
			0.107^＊^
Method of RT			
3D-CRT	46	44 (6–88)	
IMRT	19	52 (12–90	
			0.157
Concurrent chemotherapy			
CDDP	32	41 (6–90)	
TS-1		50 (20–74)	
Cmab	4	44 (24–88)	
None	2	42 (35–49)	
			0.765^＊^

OM: oral mucositis; RT: radiotherapy; CDDP: cisplatin; 3D-CRT: three-dimensional conformal radiotherapy; IMRT: intensity-modulated radiotherapy; TS-1: tegafur-gimeracil-oteracil-potassium; Cmab: cetuximab.

Prior to the start of RT, an oral care team comprising an oral surgeon and a dental hygienist examined the oral cavity and administered oral hygiene care to patients with HNC. Simultaneously, the patients were oriented regarding OM management, including instructions with regard to tooth brushing, mouth washing, oral moisturizing, nutrition, and the use of Episil® during treatment.

During the treatment, daily assessment of OM severity was conducted regarding functional disorders, symptomatic aspects, and clinical examination according to the common terminology criteria for adverse events (CTCAE) version 4.0. OM severity was graded as follows: grade 1, asymptomatic or mild symptoms, intervention not indicated; grade 2, moderate pain, not interfering with oral intake, diet modification indicated; grade 3, severe pain, interfering with oral intake; grade 4, life-threatening consequences, urgent intervention indicated; and grade 5, death.

When OM was first observed and the patients started to experience pain, we prescribed our original mouthwash containing sodium gualenate hydrate, sodium bicarbonate, and 0.2% lidocaine (Xylocaine), which was gargled 6–8 times a day. We discussed regarding Episil® use with the patients and left the decision of receiving it to the patients. Episil® was administered by one dentist (Y.K.), and the patients were instructed to use it three times per day, for at least 5–10 min before a meal. The dental hygienist explained methods of easier administration, as described earlier in the manuscript, for those patients who experienced difficulties using Episil® in this manner.

We assessed the pain relief after RT according to the WHO guidelines for the pharmacological and radiotherapeutic management of cancer pain (1). To minimize the impact of confounding factors, we confirmed the timing of all pain medications, including opioids, and ensured that there was at least a 2-h interval before commencing with Episil®. The pain-relieving effect of Episil® was confirmed by one dentist (Y.K.) after 6 h of its use through an interview with each patient and recording the patients' impressions of its use. The details of the questions are summarized in Table 3. In this study, we did not examine the duration of Episil®'s effect but focused on whether patients were able to continue using the drug for more than 6 h. Pain in the oral mucosa was assessed by a dentist (Y.K) and dental hygienist (K.I) and noted in the medical records.

**Table 3:** Questions and choices to evaluate the effectiveness and impressions of Episil® (n = 26).

Questions	Choices
Have you found Episil® to be effective in reducing pain?	Effective Not effective Favorable
How do you feel about using Episil®? (Impression)	Difficult to use Method of use needs improvement
	Impossible to use and the reason

Questions were asked 6 h after using Episil®.

All the statistical analyses were performed using the IBM SPSS version 22.0 (IBM, Armonk, NY, USA). The measurement data are expressed as the median, maximum (max), and minimum (min) values and were analyzed using the Mann–Whitney *U* test or Pearson’s Chi-square test. Fisher’s exact test was performed when more than 20% of the cases contained an expected value of 5 or less, and the chi-square test was used to calculate the other cases. The description has been corrected. The p-values calculated by Fisher’s exact test are marked with *. The statistical significance was set at *P* < 0.05.

## 3 Results

### 3.1 Patient characteristics

Table 1 lists the patients' baseline characteristics. Of the 65 patients, 26 were included in the group that used Episil® and 39 in the control group that did not use Episil®. No significant differences were observed in the baseline characteristics between the groups. The wide range of irradiation doses (30–70 Gy) was due to the inclusion of patients requiring both preoperative and post-operative irradiation. The median overall irradiation dose was 50 Gy. Additionally, the most frequently delivered dose was 50 Gy (28 patients) followed by 60 Gy (22 patients). The oral cavity was included in the irradiation area of 59 patients, and six patients were irradiated only in the neck region. Four patients were aware of pain due to OM in the pharyngeal mucosa even if irradiation was administered only to the neck and Episil® was used. No significant differences were observed regarding the RT irradiation method or concurrent chemotherapy between the groups. Two of the patients who used Episil® underwent a dose reduction of RT due to pain from OM and a deterioration in their nutritional statuses, and their RT completion rate was 97% (63/65).

### 3.2 Oral mucositis

The overall number of median days to the resolution of OM was 47 days (range, 6–90 days). The number of days to resolution of OM was not related significantly to the use of Episil® or the highest previous grade of OM during RT (Table 2); however, it was related to the irradiation dose. Compared to a lower dose, a radiation dose higher than the median total irradiation dose (50 Gy) lengthened the median healing time significantly (39 days [range, 6–57 days] vs. 53 days [range, 27–90 days]; P < 0.001) (Table 2).

### 3.3 Pain-relieving effect, duration of use, and impressions of use of Episil®

Table 4 shows the pain-relieving effects of Episil® (n = 26) confirmed within 6 h of its initial use. Of the 26 patients, 16 experienced pain relief within 6 h of Episil® use, while six reported no pain relief. No significant differences were observed with regard to the pain-relieving effect of Episil® between patients with OM grades 2 and 3.

**Table 4:** Pain-relieving effect after using Episil® (n = 26).

Pain-relieving effect	Mucositis Grade 2	Mucositis Grade 3	*P* value
Effective	7	9	
Not effective	6	4	
			0.420**∗**

The median duration of Episil® use was 30 days (range, 1–52 days). Patients who experienced pain relief at the start of Episil® use had a significantly longer duration of Episil® use (>29 days) (Table 5). In contrast, if Episil® did not improve pain at the start of use, the duration of use was ≤7 days (P < 0.001).

**Table 5:** Pain-relieving effect and duration of using Episil® (n = 26).

Pain-relieving effect	n	Days	*P* value
Not effective	10	1–7	
Effective	1	10	
Effective	15	≥29	
			<0.001∗

Table 6 shows the patients' impressions of Episil® after 6 h of use. Of the 26 patients, 10 complained of difficulty in continuing with Episil® use. Notably, these were the same patients who did not experience pain relief with Episil® at the start of its use. In addition to less relief of pain caused by OM (four patients), other reasons for difficulties in using Episil® were the inability to reach the painful area (three patients), nausea (two patients), and dislike of the smell (one patient).

**Table 6:** Impressions of Episil® use (n = 26).

Impression	n	*P* value
Favorable	7	
Difficult to use	2	
Method of use needs improvement	7	
Impossible to use	10	
		0.236

## 4 Discussion

The use of Episil® has been shown to relieve pain and improve malnutrition in patients with HNC [14]. The results of this study showed that the pain relief was not significant in patients with grade 2 and 3 OM who used Episil® (Table 3). Of the 26 patients who used Episil®, 16 experienced pain relief, which was lower than the number reported in a previous study [8]. While there have been a few reports with regard to the sole use of Episil® in patients with HNC, there has been a lack of data on the factors associated with the difficulties experienced in its use. Nevertheless, this study identified some of these factors including a lack of pain relief, an inability to reach the painful area, and a dislike of the taste and smell; the problems related to taste and smell may have been due to the oil and ethanol in the product even though patients were instructed to gargle before using Episil®. Although patients were asked about their awareness of xerostomia, no relationship was found between the presence of xerostomia and the pain-relieving effect of Episil® (data not shown). A patient-friendly and individualized manner of instruction was found to be important when teaching patients about Episil® use. In Japan, dental hygienists are often responsible for instructing patients on the use of such products. For example, Kawano et al. reported the importance of developing a patient-friendly formulation while considering different aspects, such as spray shape [15,16].

The results of this study showed that the use of Episil® had no effect on the healing time of OM caused by RT, which is consistent with a previous report [17]. The dose delivered to the oral mucosa determines the degree of OM; however, the use of concomitant chemotherapy may add to this effect [18]. In the present study—the first to assess the duration of use of Episil®—the duration of use was found to be over 30 days if it was effective in improving pain. The median healing time of OM caused by RT or CRT for HNC management was 45 days. Additionally, Episil® was used for more than 30 days if it was effective in relieving pain. Currently, health insurance in Japan prohibits the use of Episil® for over 30 days; however, the results of this study suggest that the use of Episil® for longer periods should be allowed. We found that there were many problems associated with the use of Episil®. Episil® is usually spread in the oral cavity with the tongue; however, patients with HNC have a wound in the oral cavity, which makes it difficult to apply it with the tongue and hence, individualized treatment is required. In our university hospital, dental hygienists took the lead in the treatment, and we believe that this led to the prolonged use of Episil® and the sustained pain-relieving effect.

The method and site of RT and concomitant chemotherapy may affect the patients' QOL. In particular, the impact of IMRT on the patients' QOL is controversial. The results of the meta-analysis conducted by de Felis et al. indicated that IMRT was superior to 3D-CRT, in terms of xerostomia rates [19]. However, Oba et al. reported that IMRT resulted in a progression of mucositis and worsened the patient's QOL significantly [20]. In our study, no differences were observed between the irradiation sites, methods, or chemotherapy. The incidence and healing period of OM may have differed depending on the chemotherapy administered. Further studies with a larger sample size are warranted to validate the findings of this study.

This study had some limitations. It was not a randomized controlled trial and the nutritional improvement with the use of Episil® was not evaluated, as was reported in previous studies [14]. Although there were no statistically significant differences between the group that used Episil® and the control group in terms of irradiation methods and chemotherapy, a lack of uniformity may have affected the results. Further prospective studies are necessary to determine the effect of the use of Episil® on nutritional improvement.

Although Episil® has been shown to be effective in improving the pain of OM caused by RT for patients with HNC, Episil® needs to be improved, and medical professionals are required to give careful attention to each individual patient.

## Ethics approval and consent to participate

The study was conducted in accordance with the guidelines of the Declaration of Helsinki and approved by the Institutional Review Board of the Faculty of Dentistry of Tokyo Medical and Dental University. The requirement for written informed consent from each patient was waived because of the retrospective nature of the study (approval no. D2018-016). The requirement for patient consent was waived due to the retrospective study design.

### Author contribution statement

Kanade Ito and Yuji Kabasawa: Conceived and designed the experiments; Performed the experiments; Analyzed and interpreted the data; Contributed reagents, patients, analysis tools or data; Wrote the paper.

Shiori Tokura, Itsuki Takazaawa, Hitomi Nojima., Msahiko Miura, Hiroyuki Harada and Ryoichi Yoshimura: Contributed reagents, patients, analysis tools or data.

Naomi Yoshida, Tohko Nakanishi, Kikue Akiyama, Yuki Onuma and Toshiko Adachi; Contributed reagents and patients. of the manuscript.

## Data availability statement

Data will be made available on request.

## Declaration of competing interest

The authors declare that they have no known competing financial interests or personal relationships that could have appeared to influence the work reported in this paper
